# Comparing the Perioperative and Oncological Outcomes of Open Versus Minimally Invasive Inguinal Lymphadenectomy in Penile Cancer: A Systematic Review and Meta-Analysis

**DOI:** 10.3390/cancers17183035

**Published:** 2025-09-17

**Authors:** Yu Guang Tan, Khi Yung Fong, Nathanael Kai-Jun Goh, Alvin YM Lee, Kae Jack Tay, John SP Yuen, Michael R. Abern, Kenneth Chen

**Affiliations:** 1 Department of Urology, Singapore General Hospital, Singapore 169854, Singaporekenneth.chen@singhealth.com.sg (K.C.); 2 Department of Urology, Duke University School of Medicine, Durham, NC 27710, USA

**Keywords:** penile cancer, inguinal lymph node dissection, minimally invasive surgery, laparoscopy, robotic surgery

## Abstract

Background: Long-term survival in penile cancer is dependent on the presence and extent of lymph node metastases. Historically, inguinal lymph node dissection (ILND) has been performed via an open approach (O-ILND). More recently, minimally invasive surgical alternatives (MIS-ILND) such as video-endoscopic and robot-assisted ILND have emerged. This review aims to compare the (1) perioperative outcomes, (2) complication rates, and (3) oncological efficacy between O-ILND and MIS-ILND. Methods: We conducted a PRISMA-compliant meta-analysis including studies comparing O-ILND versus MIS-ILND for penile cancer. Outcomes were pooled in random-effects meta-analyses. Results: Sixteen articles comprising 1054 patients were analysed. There was an observed trend towards longer operative time for the MIS-ILND approach (mean difference 28 min; 95% CI −2 to 58 min, *p* = 0.06), particularly with the robotic-assisted technique. Total LN yield (mean 12.3, mean difference 0.3, 95% CI −0.3 to 0.9, *p* = 0.13), and positive LN (RR 0.98, 95% CI 0.88–1.10, *p* = 0.75) were similar between groups. MIS-ILND significantly reduced complication rates for both minor (RR: 0.65, 95% CI 0.45–0.94, *p* = 0.02) and major complications (RR: 0.25, 95% CI 0.12–0.53, *p* = 0.002). Particularly, there was also lower wound infection rate with MIS-ILND (RR: 0.43, 95% CI 0.22–0.82, *p* = 0.02), corresponding to a shorter hospital stay of average 4 days (MD −4, 95% CI −6–−2, *p* = 0.05). Rates of skin/flap necrosis, lymphedema, lymphocele, and drainage time did not differ significantly. Local groin recurrence and overall survival did not differ between approaches. Conclusions: MIS-ILND is associated with fewer perioperative complications and shorter hospitalisation without compromising oncologic outcomes. These findings support its broader adoption, particularly in high-volume centres with appropriate surgical expertise.

## Simple Summary

Penile cancer is a rare but disfiguring and destructive genitourinary cancer. It carries a high propensity for early lymph node metastasis. While inguinal lymph node dissection (ILND) is both prognostic and therapeutic in the management of penile cancer, it carries significant morbidities and wound complications when performed in conventional open manner. The advent of minimally invasive surgeries (MIS) has been shown to reduce perioperative complications but has raised concerns regarding its oncological efficacy. This study provides a comprehensive review comparing open vs. MIS ILND and demonstrates that MIS ILND can achieve comparable oncological outcomes while significantly reducing perioperative complications.

## 1. Introduction

Penile carcinoma is a rare genitourinary malignancy which accounts for less than 1% of all male cancers. Despite its rarity, it carries a disproportionate disease burden due to its propensity for delayed presentation [1], aggressive growth, and early lymphatic spread. Management of penile cancer also often carries significant physical and psychological morbidity, such as disfigurement, impairment of urinary and sexual function, and long-term psychosocial consequences, including depression and reduced quality of life [2,3].

The presence and extent of lymph node (LN) metastases remain the most important prognostic factors for long-term survival [4]. Lymphatic metastasis in penile cancer occurs in a predictable and stepwise manner, following the route of anatomical drainage from the primary tumour to the superficial and then deep inguinal LNs, followed by the ipsilateral pelvic LNs. Inguinal lymph node dissection (ILND) for penile cancer is diagnostic and prognostic, and it is amongst the few genitourinary cancers proven to have a therapeutic benefit [5]. Historically, ILND has been performed via an open approach (O-ILND). However, O-ILND is associated with substantial morbidity, notably wound infection, skin necrosis, lymphoedema, lymphocele formation, and seroma [6]. To reduce these complications, minimally invasive surgical (MIS) alternatives such as video-endoscopic ILND (VEIL) and robot-assisted VEIL (RA-VEIL) have emerged recently. These MIS techniques aim to replicate the oncologic completeness of open dissection in achieving good nodal yield while minimising soft tissue trauma and wound-related complications. Given these evolving surgical practices and the uncertainty regarding the comparative oncological outcomes, we conducted a systematic review and meta-analysis to evaluate the perioperative outcomes, complication rates, and oncological efficacy between O-ILND and MIS-ILND in penile cancer.

## 2. Methods

### 2.1. Literature Search

This systematic review and meta-analysis were performed in line with the Preferred Reporting Items for Systematic Reviews and Meta-Analyses (PRISMA) Guidelines and registered with PROSPERO (CRD420251111349).

An electronic literature search from database inception till 31 March 2025 was conducted by two independent investigators on PubMed, EMBASE, and Scopus for relevant articles, without language restrictions (Supplementary Table S1). Bibliographies of each included study were screened, and a search on Google Scholar using the first and last author of each included study was conducted to ensure inclusion of all relevant studies. Abstracts and full texts were reviewed by two independent investigators (Y.G.T. and K.Y.F.), with conflicts resolved by a third investigator (K.C.).

### 2.2. Inclusion and Exclusion Criteria

We included prospective or retrospective studies directly comparing O-ILND versus MIS-ILND (laparoscopic or robotic) for penile cancer or where majority of the patients had penile cancer. The intervention arm was MIS-ILND and the control arm was O-ILND. Case reports, reviews, conference abstracts, and systematic reviews were excluded. Eligible studies had to include at least one of the following outcomes of interest: operative/perioperative outcomes, complication rates, and oncological outcomes. A standardised data collection template with predefined data fields including study characteristics, patient demographics, and outcomes was used for data extraction by two independent investigators.

### 2.3. Quality Assessment and Risk of Bias

Risk of bias was assessed using the Newcastle–Ottawa Scale for cohort studies (Supplementary Table S2).

### 2.4. Meta-Analysis

The primary outcomes were broadly divided into three categories: (1) operative/perioperative outcomes (total operative time, blood loss, lymph node yield (LNY), positive nodal count, length of stay (LOS), and time to drain removal); (2) complication rates (Clavien–Dindo classification, wound infection, and skin/flap necrosis); and (3) oncological outcomes (overall recurrence, local groin recurrence, and overall survival (OS)).

For continuous outcomes, means and standard deviations (SDs) between OILND and MIS-ILND were pooled in random-effects meta-analyses to determine mean differences (MD) and 95% confidence intervals (95% CI). For binary outcomes, respective numbers of patients per arm and patients with the outcome in question were pooled in random-effects meta-analyses to determine incidence odds ratios (ORs) and 95% CI. Prior to meta-analysis, missing means and SDs were derived from medians and interquartile ranges according to methods proposed by Hozo et al. [7] and Wan et al. [8]. Network meta-analysis of robotic (RA-VEIL) versus laparoscopic (VEIL) versus open ILND was also conducted, using a Frequentist random-effects model. A forest plot, league table and league diagram were generated.

Heterogeneity was considered low, moderate, or considerable for I^2^ values <40%, 40–75%, and >75%, respectively. Funnel plot symmetry was visually assessed for publication bias [9]. Certainty of evidence was assessed using the GRADE (Grading of Recommendations Assessment, Development and Evaluation) framework [10]. All statistical analyses were carried out using RStudio (version 4.3.0, Posit Software, Boston, MA, USA), with *p* < 0.05 regarded to indicate statistical significance.

## 3. Results

### 3.1. Study Selection

**Figure 1:**
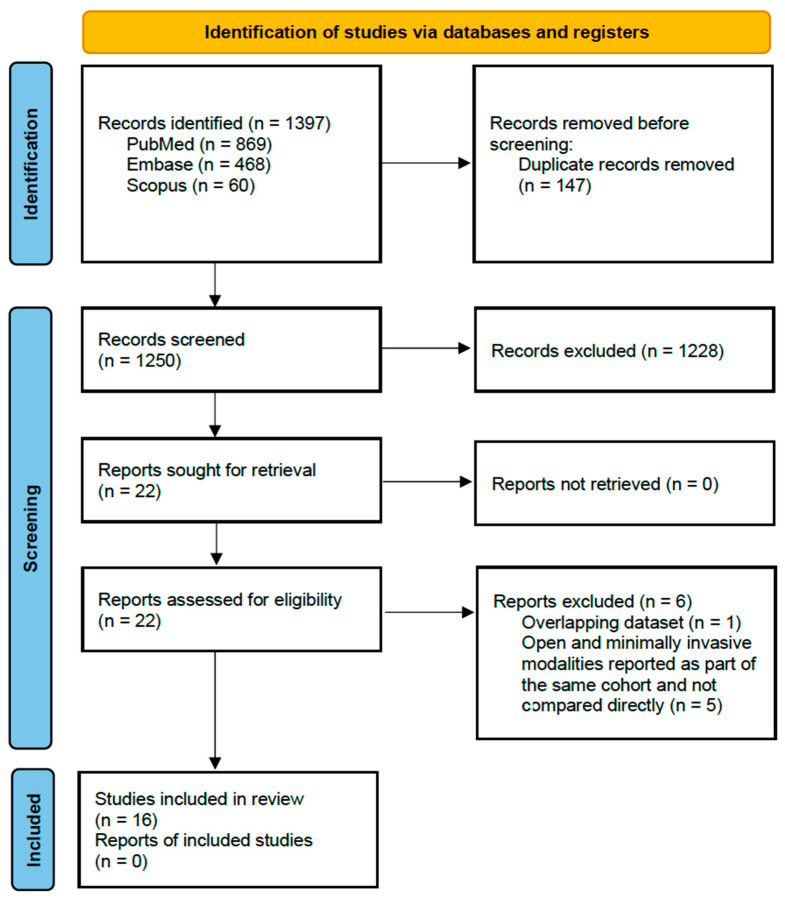
PRISMA flowchart of included studies.

The search strategy yielded 1397 studies. After removal of 147 duplicates, 1250 studies underwent title and abstract screening. A total of 22 manuscripts were identified for full-text review. Sixteen studies met the inclusion criteria and were analysed (Table 1, Figure 1). Most studies were published after 2017, with only Tobias-Machado [11] being published in 2007. In total, there were 1054 patients, and the total number of patients varied in each study, ranging from 19 to 206. Twelve studies compared O-ILND to VEIL (laparoscopic), while four studies compared O-ILND to robotic-assisted surgery (RA-VEIL). Most studies were retrospective cohort studies comparing O-ILND and MIS-ILND in separate patient cohorts, but notably Tobias-Machado [11], Yadav [12] and Falcone [13] were designed as prospective randomised controlled studies, with the patient acting as his own control (OILND on one side, MIS-ILND on contralateral side).

The mean age of patients was 54 years old (IQR 48–70). The majority of studies recruited only patients with penile squamous cell carcinoma (SCC), with the exception of Schwentner et al. [14] who also included a small proportion of penile melanoma (29%). Nine studies reported the treatment for the primary penile cancer, with amputative surgeries (glansectomy, partial and total penectomy) being the most commonly performed. The indication for ILND was mostly therapeutic in all except one study [11], which was performed for prophylactic intent based on high risk penectomy pathological features such as pT1, high grade, and presence of lymphovascular invasion. Notably, four studies [15,16,17,18] recruited patients with clinically palpable and enlarged inguinal nodes, whereas three studies reported prior dynamic sentinel node biopsy or fine needle aspiration cytology to confirm nodal disease prior to therapeutic ILND [19,20,21].

### 3.2. Operative/Perioperative Outcomes

**Figure 2:**
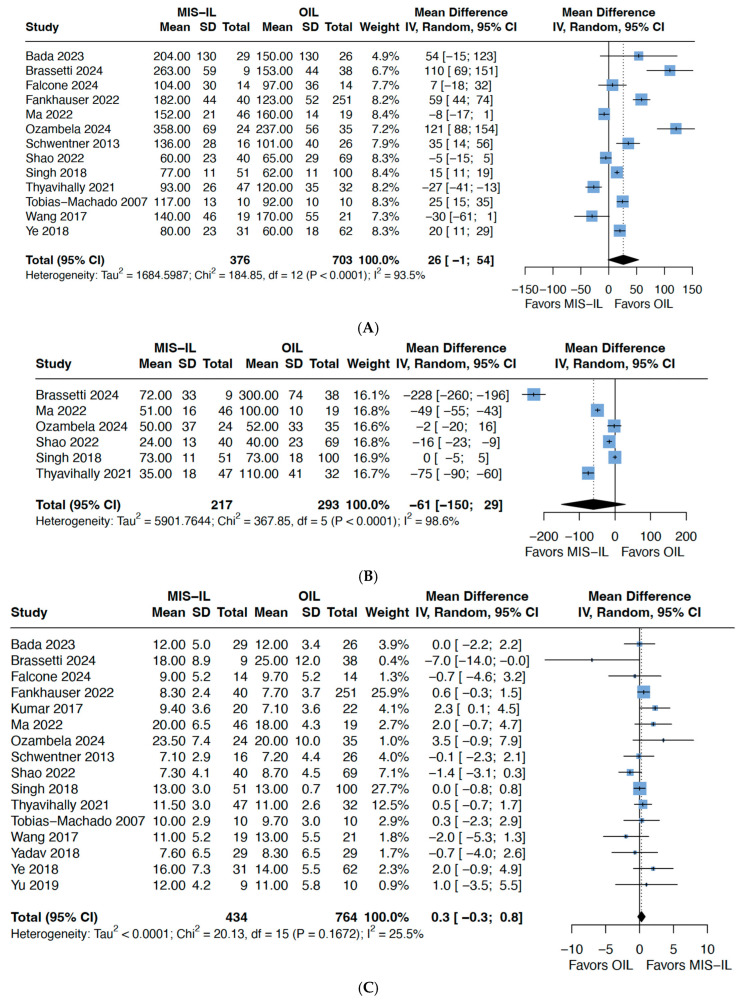

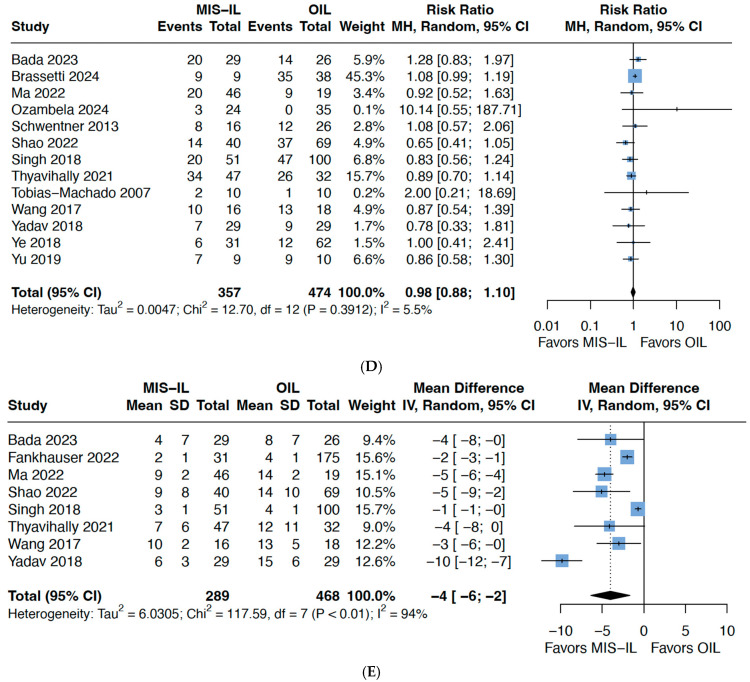
Forest plots comparing O-ILND versus MIS-ILND for (**A**) operative time in minutes; (**B**) estimated blood loss in ml; (**C**) lymph node yield; (**D**) groins with positive inguinal lymph nodes; and (**E**) length of stay in days.

Across twelve studies, there was an observed trend towards longer operative time for the MIS-ILND approach (mean difference 28 min; 95% CI −2 to 58 min, *p*: 0.06, I^2^: 94%), albeit there was significant heterogeneity (Figure 2A). In a three-arm Bayesian network meta-analysis comparing O-ILND, VEIL, and RA-VEIL, RA-VEIL had a longer operative time, whereas there was no significant difference between VEIL and O-ILND (Supplementary Figures S1 and S2, Supplementary Table S3). Estimated blood loss was not significantly different between O-ILND and MIS-ILND (MD −61; 95% CI −150 to 29 mL, *p* = 0.14) (Figure 2B). Across all fifteen studies, there was no significant difference in the mean LNY for O-ILND (mean: 12.3, range 7.1–25.0) and MIS-ILND (mean 12.3, range 7.1–23.5) (mean difference 0.3, 95% CI −0.3 to 0.9, *p* = 0.13) (Figure 2C), with the exception of Brassetti [15] who observed an average of seven more lymph nodes per patient harvested for O-ILND (25 nodes, IQR: 17–33) than in MIS-ILND (18 nodes, IQR: 12–24). The proportion of patients or inguinal sides with positive lymph nodes was again similar (RR 0.98, 95% CI 0.88–1.10, *p* = 0.75) (Figure 2D). Across eight studies, a shorter LOS of an average of 4 days was observed in favour of MIS-ILND (MD −4, 95% CI −6–−2, *p* = 0.05) (Figure 2E). There was also no difference in the time to drain removal (MD 0 days; 95% CI −4 to 5 days, *p* = 0.91), although six of the ten studies demonstrated MIS-ILND had a significant shorter time to drain removal (Supplementary Figure S3).

### 3.3. Complications

**Figure 3:**
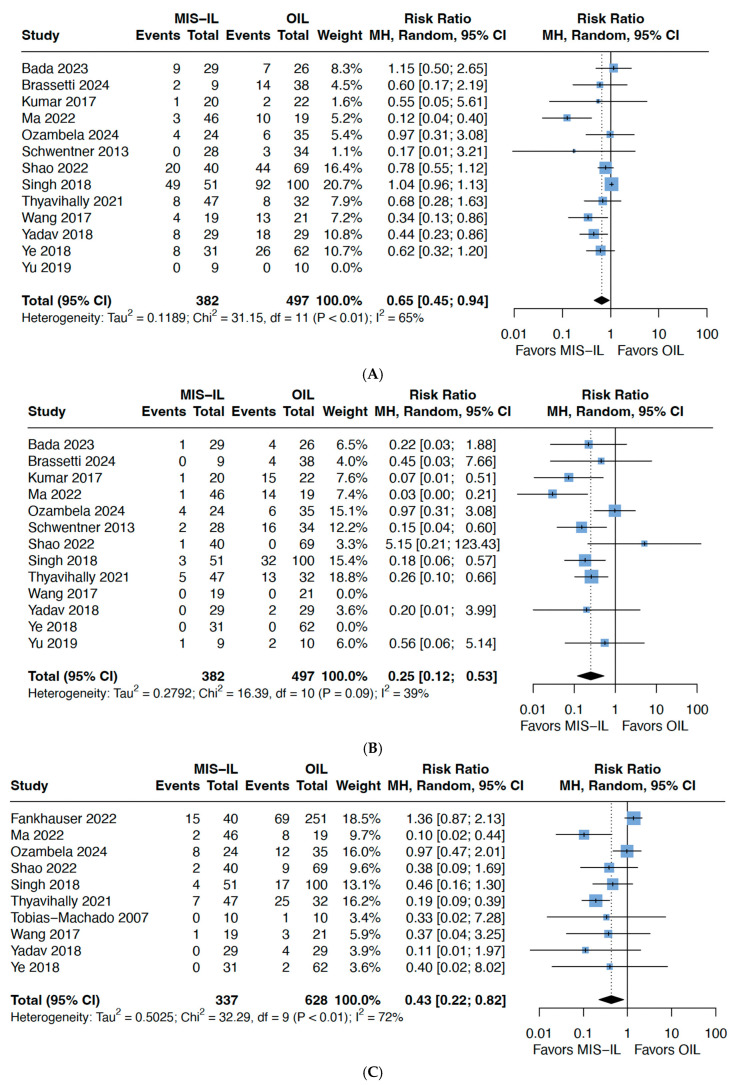
Forest plots comparing O-ILND versus MIS-ILND for (**A**) minor complications (Clavien–Dindo Grade 1–2); (**B**) major complications (Clavien–Dindo Grade > 2); and (**C**) wound infection.

Thirteen studies used the Clavien–Dindo (CD) classification to classify and compare complications for both MIS-ILND and O-ILND and consistently revealed that MIS-ILND had a significantly lower complication rate. MIS-ILND had better outcomes for minor complications (CD 1–2) (HR: 0.65, 95% CI 0.45–0.94, *p* = 0.02, I^2^: 65%) (Figure 3A), and the benefit of the MIS approach was also observed for major complications (CD > 2) with low heterogeneity (HR: 0.25, 95% CI 0.12–0.53, *p* = 0.002, I^2^: 39%) (Figure 3B). Specifically, there were lower rates of wound infection for the MIS approach (HR: 0.43 95% CI 0.22–0.82, *p* = 0.02, I^2^: 72%) (Figure 3C), which contributed to the abovementioned shorter LOS. Other studied complications such as skin/flap necrosis, lymphedema, lymphocele, and deep vein thrombosis were similar in both groups (Supplementary Figures S4–S7).

### 3.4. Oncological Outcomes

**Figure 4:**
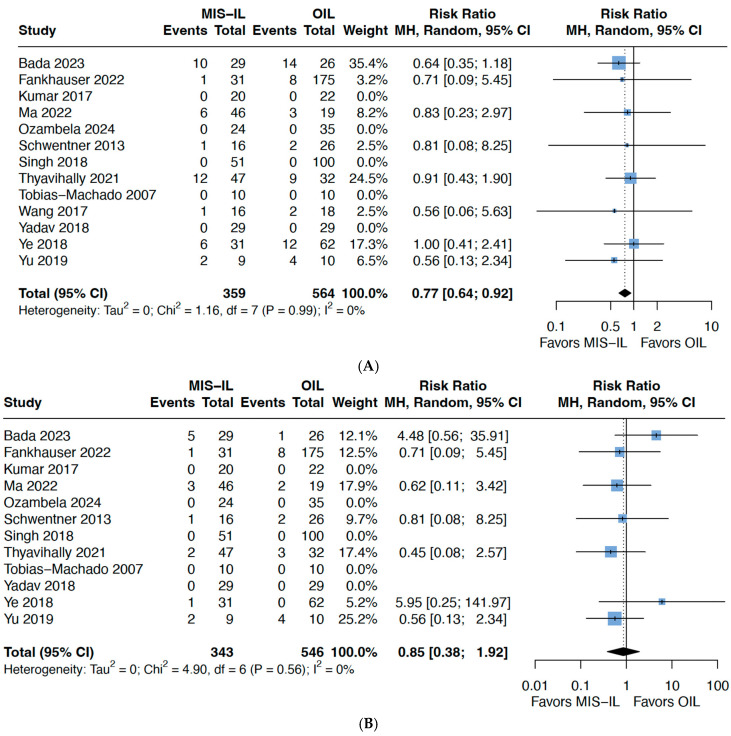
Comparing O-ILND versus MIS-ILND for (**A**) overall recurrence; (**B**) local groin recurrence.

The follow-up period varied across studies, ranging from 12 to 96 months, with most studies reporting follow up beyond 24 months. Across seven studies, overall recurrence favoured MIS-ILND (HR 0.77, 95% CI 0.64–0.92, *p* = 0.01) (Figure 4A), but there was no difference in the local groin recurrence (HR: 0.85, 95% CI 0.38–1.92, *p* = 0.65) (Figure 4B). Overall survival was reported in several studies, but no data of exact event numbers could be obtained for pooling. Nonetheless, studies reporting this outcome all found no significant differences between O-ILND and MIS-ILND [15,22,23,24]. In the study by Ye [25], *p*-values were not reported but mortality rate was 16% in both VEIL and O-ILND. In the randomised controlled trials (RCTs) by Tobias-Machado [11], Yadav [12], and Falcone [13], there was no appreciable comparison, since both techniques were performed on the same patient. Altogether, included studies seemed to suggest that OS is not significantly different between O-ILND and MIS-ILND. Certainty of evidence was mostly low to moderate [Table 2]. Funnel plots did not show any obvious publication bias (Supplementary Figures S8–S22).

## 4. Discussion

Penile carcinoma is a rare but disproportionately destructive genitourinary malignancy, often causing significant physical and psychological morbidity, from both the disease itself and the surgical treatment. The increasing interest in MIS-ILND gives a greater impetus to analyse its oncological efficiency as compared to O-ILND before urologists can safely adopt this technique. Most clinical studies are hampered by the relatively small sample sizes; therefore, pooling of data from published cohort studies can provide greater understanding of the clinical outcomes. There are few published meta-analyses in this regard Hu et al. [27] compared VEIL with O-ILND and observed a reduction in LOS and drainage time but a lower number of LNY with VEIL, raising concerns about the latter’s oncological inferiority. However, the study was published much earlier and could have reflected the infancy stages and required learning curve for MIS-ILND. Patel et al. [28] extended this scope to include studies using both VEIL and RA-VEIL techniques, but the corresponding small sample size and consequent poor study quality raises questions about the accuracy of their findings. Our review extends this work by including more recently published studies not captured in either review, expanding the sample size and thereby improving the precision of effect estimates. The contemporaneous studies predominantly published in the past five years also reflect the refinement of MIS-ILND to achieve good nodal dissection. Across 16 studies involving over 1000 patients, this systematic review demonstrated that while MIS-ILND could be associated with longer operative times, it demonstrated significantly lower postoperative complications, particularly wound-related issues, while achieving comparable oncologic outcomes as measured by similar LNY and recurrence rates. The longer operative times importantly did not translate into increased intraoperative morbidity or worse outcomes and in fact, achieved a shorter LOS. These findings are corroborated by other studies [28,29,30], and suggest that MIS-ILND offers a clinically meaningful advantage in perioperative safety without compromising oncologic efficiency.

Wound infections constitute a major morbidity of ILND. The EAU guidelines [3] report wound infection rates of 2–43% and skin necrosis rate of 3–50%. This systematic review observed similar statistics, with wound infection rates ranging from 3% to 78% (median: 15.5%, weighted average: 24%) and skin necrosis rates from 0% to 44% (median: 12.5%, weighted average: 20%). This would have led to longer hospital stay, drainage duration, more resources required for wound care management, and most importantly, a potential delay in adjuvant chemotherapy for some patients. Many incisional modifications have been suggested for O-ILND, such as the transverse (S-shape), vertical single oblique and dual skin bridge (Fraley) incisions [31,32], to reduce skin-related morbidities. Ray et al. [33] found the Fraley incision to be highly effective in minimising wound postoperative wound complications while maintaining optimal early oncological outcomes, but this was not observed by Shao et al. [23]. Bertheuil et al. [34] similarly reported no significant difference in wound-related complications when comparing S-shaped and vertical incisions. Many factors beyond the optimal skin incision influence the risk of developing wound infections, ranging from patient (age, comorbidities, obesity, smoking, preoperative skin hygiene) to procedural factors (operative time, operative approach, extent of tissue trauma and devascularisation). This review paper highlighted the significant reduction in complication rates with an MIS approach, which has been well observed in intra-abdominal and other genitourinary cancers. More importantly, the observed benefit was greater in major compared to minor complications with low heterogeneity across the studies. Singh et al. [17] reported similar overall complication rates between O-ILND and RA-VEIL, but a significant reduction in major complications in the RA-VEIL cohort (2% vs. 17%), notably a lower skin necrosis and severe lymphedema. The potential benefit of lower rates of lymphedema, lymphocele, and deep vein thrombosis observed in individual studies [27,35,36,37] was not observed in the pooled analysis. We believe these complications could be related to the adequacy of lymph node removal and extent of saphenous vein preservation rather than the surgical approach itself. Finally, in selected patients where ipsilateral pelvic lymph node dissection is required for advanced disease, the MIS-ILND can avoid the need of an additional abdominal incision.

Prior to advocating the widespread adoption of MIS for ILND, it is important to evaluate the patient selection and oncological outcomes for either approach. The sixteen studies analysed in this review were similar in terms of the patient profile. In particular, four studies recruited patients with clinically enlarged and palpable inguinal lymphadenopathy, to reflect the utility of MIS-ILND in advanced disease. This challenges previous misconception that MIS-ILND is predominantly reserved for early disease with subclinical metastasis. Previous studies have proposed that a minimal LNY > 7 is necessary to reflect a reliable oncological procedure and quality assessment [37,38,39], and this review has demonstrated similar LNY for either MIS-ILND and O-ILND, with a mean yield of 12 nodes. Individual studies [20,29,40] had even demonstrated a higher nodal count with MIS-ILND, as a result of the improved surgical visualisation and technical expertise. Altogether, the comparable LNY observed in MIS-ILND supports the observation of a similar local recurrence rate across all studies.

There are some inherent limitations of this review paper. Firstly, most studies were retrospective cohort studies with small sample size, which presented significant heterogeneity in the studied demographics. The indication for ILND (prophylactic or therapeutic) varied and the extent of ILND was not explicitly stated in most studies, although there was no significant difference in the LNY or positive nodes across each study. Some of the outcome measurements such as lymphocele, lymphedema, and flap necrosis were not clearly defined and raised ambiguity during analysis. The extent of saphenous vein preservation, which is an important factor in reducing postoperative lymphedema and DVT, was not elaborated in most studies. Other key outcomes such operative blood loss and LOS also exhibited high heterogeneity which may reflect the learning curve for MIS-ILND and the surgeon’s experience in this relatively uncommon procedure. Nonetheless, in the three RCT-designed studies, where patients undergo O-ILND on one side and MIS-ILND on the contralateral thigh, similar findings of lower complication rates with comparable oncological outcomes were observed. Lastly, while most studies reported similar local groin recurrence and overall survival, the follow-up durations were short and inconsistently reported, meaning that conclusions regarding long-term oncologic outcomes should be interpreted with caution.

## 5. Conclusions

This systematic review demonstrates that MIS-ILND offers perioperative advantages of fewer complications, reduced wound morbidity, and shorter hospitalisation. Importantly, these benefits are achieved without compromising oncologic outcomes, including lymph node yield, recurrence, or survival. As surgical expertise and technology advance, MIS-ILND has the potential to become the preferred standard for managing inguinal nodal disease in penile cancer, especially in high-volume centres with the requisite surgical expertise.

## Figures and Tables

**Table 1:** Baseline characteristics of included studies.

Study, Year	Study Design	Arm	Number of Patients	Same Patients	Age	Smoker, %	DM, %	BMI, kg/m^2^	Follow-Up, Months	Histology, %	Index Penile Surgery, %	Indication for ILND
Tobias-Machado 2008 [11]	Prospective randomised	OILND	10	Yes (one on each side; 20 groins)	48 (39–60)	NR	NR	NR	19 (12–31)	SCC	Penile amputation	Prophylactic (cN0)
		VEIL							19 (12–31)	SCC		
Yadav 2018 [12]	Prospective randomised	OILND	29	Yes (one on each side; 58 groins)	52.4	NR	NR	NR	14 (7–28)	SCC	Partial or total	Mixed (therapeutic and prophylactic)
Schwentner 2013 [14]	Retrospective	OILND	26 (34 cases)	No	59	NR	NR	NR	56 (2–87)	Penile 64%, melanoma 29%	NR	NR
		VEIL	16 (28 cases)		63	NR	NR	NR				
Falcone 2024 [13]	Prospective randomised	OILND	14	Yes (one on each side; 28 groins)	63 (57–69)	14	NR	29 (26–32)	14 (12–17)	NR	NR	Mixed (therapeutic and prophylactic)
		VEIL	14									
Brassetti 2024 [15]	Retrospective	OILND	38	No	58 (53–67]	NR	34	26 (24–30)	96	SCC	Partial or total	Therapeutic (cN2)
		RA-VEIL	9		68 (52–73)	NR	33	26 (25–29)		SCC		
Ma 2022 [16]	Retrospective	OILND	19	No	52 ± 13	NR	NR	26 (23–28)	48 (34–60)	SCC	NR	Therapeutic (cN1/2)
		S-VEIL	24		56 ± 9.3	NR	NR	24 (22–26)	36 (30–42)	SCC		
		D-VEIL	22		55 ± 11	NR	NR	23 (22–25]	34 (26–47)	SCC		
Singh 2018 [17]	Retrospective	OILND	100	No	54 (45–64)	37	30	25 (23–29]	40 (26–59)	SCC	NR	Therapeutic (cN1/2)
		RA-VEIL	51		58 (50–68)	41	33	26 (23–31)	41 (28–57)	SCC		
Yu 2019 [18]	Retrospective	OILND	10	No	55 ± 13	NR	NR	27 (22–30)	53 (25–70)	SCC	NR	Therapeutic (cN1–3)
		RA-VEIL	9		50 ± 7.2	NR	NR	27 (22–33)	25 (15–29)	SCC		
Fankhauser 2022 [19]	Retrospective	OILND	251	No	63 ± 13	NR	13	29 (19–47)	21 (8–54)	SCC 85, basaloid 10, sarcomatoid 3%	Circumcision/WLE 17, glansectomy 15, partial 48, total 18	Therapeutic (Positive FNAC/DSNB)
		VEIL	40					29 (19–47)	12 (4–17)			
Kumar 2017 [20]	Retrospective	OILND	22	No	70	NR	NR	NR	71 (30–99)	NR	NR	Therapeutic (Positive DSNB)
		VEIL	20		66	NR	NR	NR	16 (4–35)	NR		
Ozambela 2024 [21]	Retrospective	OILND	35	No	68 (51–74)	54	NR	31 (26–34)	33	SCC	Partial 66, radical 14, WLE 20	Therapeutic (Positive FNAC/DSNB)
		RA-VEIL	24		65 (54–71)	67	NR	30 (28–38)	40	SCC	Partial 83, radical 13, WLE 4	
Bada 2023 [22]	Retrospective	OILND	26	No	59 ± 9.9	62	61	26 (23–31)	60	SCC	Glansectomy 35, partial 31, total 7.7	Mixed (therapeutic and prophylactic)
		VEIL	29		62 ± 12	24	34	26 (23–29)		SCC	Glansectomy 24, partial 17, total 17	
Shao 2022 [23]	Retrospective	OILND	69	No	51 ± 13	57	NR	NR	43 (15–87)	SCC	Partial or radical penectomy	Mixed (therapeutic and prophylactic)
		VEIL	40		51 ± 13	48	NR	NR		SCC		
Thyavihally 2021 [24]	Retrospective	OILND	32	No	60 (54–62)	50	NR	25 (24–28)	51 (26–76)	SCC	Partial 66, total 34	Mixed (therapeutic and prophylactic)
		VEIL	47		58 (50–62)	34	NR	26 (24–28)	42 (21–62)	SCC	Partial 60, total 40	
Ye 2018 [25]	Retrospective	OILND	62	No	54 (33–82)	NR	NR	23 (16–34)	22 (14–47)	NR	NR	Mixed (therapeutic and prophylactic)
		VEIL	31		54 (34–79)	NR	NR	24 (17–32)		NR		
Wang 2017 [26]	Retrospective	OILND	18 (3 bilateral)	No	59 ± 8.4	NR	NR	NR	12	SCC	Penile amputation or radical resection	Mixed (therapeutic and prophylactic)
		VEIL	16 (3 bilateral)		54 ± 9.9	NR	NR	NR		SCC		

OILND, open inguinal lymph node dissection; VEIL, video-endoscopic inguinal lymphadenectomy (S-VEIL = single laparoscopy; D-VEIL = double laparoscopy); RA-VEIL, robot-assisted video-endoscopic inguinal lymphadenectomy; DM, diabetes mellitus; BMI, body mass index; WLE, wide local excision; SCC, squamous cell carcinoma; NR, not reported; FNAC, fine needle aspiration cytology; DSNB, dynamic sentinel node biopsy.

**Table 2:** Certainty of evidence across studies.

Outcome	Relative Effect (95% CI)	N (Studies)	*p*-Value	Heterogeneity (%)	Certainty of Evidence (GRADE)
Recurrence (overall)	RR 0.77 (0.64–0.92)	593 (7 studies)	0.01	0	⊕⊕⊕⊝ Moderate *
Recurrence (local)	RR 0.85 (0.38–1.92)	559 (7 studies)	0.65	0	⊕⊕⊕⊝ Moderate *
Total operative time	MD 26 (−1; 54)	1079 (13 studies)	0.06	94	⊕⊕⊝⊝ Low *^
Estimated blood loss	MD −61 (−150; 29)	510 (6 studies)	0.14	99	⊕⊕⊝⊝ Low *^
Lymph node yield	MD 0.3 (−0.3; 0.8)	1198 (16 studies)	0.31	26	⊕⊕⊕⊝ Moderate *
Lymph node positivity	RR 0.98 (0.88–1.10)	831 (13 studies)	0.75	5.5	⊕⊕⊕⊝ Moderate *
Clavien–Dindo 1–2 complications	RR 0.65 (0.45–0.94)	860 (12 studies)	0.02	65	⊕⊕⊝⊝ Low *^
Clavien–Dindo 3–4 complications	RR 0.25 (0.12–0.53)	746 (11 studies)	0.002	39	⊕⊕⊕⊝ Moderate *
Wound infection	RR 0.43 (0.22–0.82)	965 (10 studies)	0.02	72	⊕⊕⊝⊝ Low *^
Lymphedema	RR 0.77 (0.43–1.39)	1051 (12 studies)	0.36	62	⊕⊕⊝⊝ Low *^
Lymphocele/seroma	RR 0.96 (0.71–1.30)	1104 (14 studies)	0.10	34	⊕⊕⊕⊝ Moderate *
Deep venous thrombosis	RR 0.31 (0.07–1.41)	629 (3 studies)	0.10	0	⊕⊕⊕⊝ Moderate *
Skin/flap necrosis	RR 0.40 (0.12–1.33)	599 (7 studies)	0.11	56	⊕⊕⊝⊝ Low *^
Length of hospital stay	MD −4 (−6; −2)	757 (8 studies)	0.005	94	⊕⊕⊝⊝ Low *^
Time to drain removal	MD −1 (−6; 4)	903 (10 studies)	0.79	89	⊕⊕⊝⊝ Low *^

* Downgraded one level due to use of data from nonrandomized and nonmatched studies. ^ Downgraded one level due to moderate or high heterogeneity. MD, mean difference; RR, risk ratio.

## Data Availability

The authors confirm that the data supporting the findings of this study are published within the main article and the Supplementary Materials.

## Supplementary Materials

The following supporting information can be downloaded at https://www.mdpi.com/article/10.3390/cancers17183035/s1, Figure S1: Forest plot of Bayesian network meta-analysis of operative time; Figure S2: Network graph of Bayesian network meta-analysis of operative time; Figure S3: Forest plot comparing O-ILND versus MIS-ILND for time to drain removal; Figure S4: Forest plot comparing O-ILND versus MIS-ILND for skin/flap necrosis; Figure S5: Forest plot comparing O-ILND versus MIS-ILND for lymphedema; Figure S6: Forest plot comparing O-ILND versus MIS-ILND for lymphocele; Figure S7: Forest plot comparing O-ILND versus MIS-ILND for deep vein thrombosis; Figure S8: Funnel plot for operative time (min); Figure S9: Funnel plot comparing estimated blood loss (mL); Figure S10: Funnel plot comparing lymph node yield; Figure S11: Funnel plot comparing groins with positive inguinal lymph nodes; Figure S12: Funnel plot comparing length of stay (days); Figure S13: Funnel plot comparing minor complications (Clavien-Dindo Grade 1–2); Figure S14: Funnel plot comparing major complications (Clavien-Dindo Grade > 2); Figure S15: Funnel plot wound infection; Figure S16: Funnel plot comparing overall recurrence; Figure S17: Funnel plot comparing local groin recurrence; Figure S18: Funnel plot comparing time to drain removal (days); Figure S19: Funnel plot comparing skin/flap necrosis; Figure S20: Funnel plot comparing lymphedema; Figure S21: Funnel plot comparing lymphocele; Figure S22: Funnel plot comparing deep vein thrombosis; Table S1: Full search phrases for the respective databases; Table S2: Risk of bias assessed using Newcastle-Ottawa Scale; Table S3: Bayesian network meta-analysis of operative time in minutes.
