# Supplementary material for: Comparing the Perioperative and Oncological Outcomes of Open Versus Minimally Invasive Inguinal Lymphadenectomy in Penile Cancer: A Systematic Review and Meta-Analysis
Source: Cancers (Basel). 2025 Sep 17;17(18):3035. doi: 10.3390/cancers17183035 (PMC12468625; doi:10.3390/cancers17183035)
Supplement: Supplementary file 1 [file cancers-17-03035-s001.zip › cancers-3798622-supplementary.pdf]

Supplementary Materials

Comparing the Perioperative and Oncological Outcomes of Open Versus Minimally Invasive Inguinal Lymphadenectomy in Penile Cancer: A Systematic Review and Meta-Analysis

Yu Guang Tan [1,*], Khi Yung Fong [1], Nathanael Kai-Jun Goh [1], Alvin YM Lee [1], Kae Jack Tay [1], John SP Yuen [1], Michael R. Abern [2] and Kenneth Chen [1]

Table S1. Full search phrases for the respective databases.

PubMed	869 articles
(penis OR penile) AND (cancer OR carcinoma) AND (lymph node dissection OR lymphadenectomy) NOT (systematic[sb] OR Editorial[pt] OR Comment[pt] OR Meta-Analysis[pt] OR Case Reports[pt] OR Review[pt])	
**Embase**	**468 articles**
(penis OR penile) AND (cancer OR carcinoma) AND (lymph AND node AND dissection OR lymphadenectomy) AND (laparoscopic OR robot OR open OR endoscopic OR endoscopy OR veil OR 'minimally invasive surgery' OR mis) NOT ([animals]/lim NOT [humans]/lim) NOT ([editorial]/lim OR [erratum]/lim OR [letter]/lim OR [note]/lim OR [review]/lim OR [short survey]/lim)	
**Scopus**	**60 articles**
TITLE-ABS-KEY ((penis OR penile) AND (cancer OR carcinoma) AND (lymph node dissection OR lymphadenectomy)) AND NOT (SRCTYPE (b) OR SRCTYPE (k) OR SRCTYPE (p) OR SRCTYPE (r) OR SRCTYPE (d) OR DOCTYPE (ab) OR DOCTYPE (bk) OR DOCTYPE (ch) OR DOCTYPE (bz) OR DOCTYPE (cp) OR DOCTYPE (cr) OR DOCTYPE (ed) OR DOCTYPE (er) OR DOCTYPE (le) OR DOCTYPE (no) OR DOCTYPE (pr) OR DOCTYPE (rp) OR DOCTYPE (re) OR DOCTYPE (sh))	

Table S2. Risk of bias assessed using Newcastle-Ottawa Scale.

	Selection	Comparability	Outcome	Total quality score
Bada 2023 [1]	***	*	**	6
Brassetti 2024 [2]	***	*	**	6
Falcone 2024 [3]	****	**	**	8
Fankhauser 2022 [4]	***	*	**	6
Kumar 2017 [5]	***	*	*	5
Ma 2022 [6]	***	*	**	6
Ozambela 2024 [7]	***	*	**	6
Schwentner 2013 [8]	**	*	**	5
Shao 2022 [9]	***	*	**	6
Singh 2018 [10]	***	*	**	6
Thyavihally 2021 [11]	***	*	**	6
Tobias-Machado 2007 [12]	***	**	**	7
Wang 2017 [13]	***	*	**	6
Yadav 2018 [14]	**	*	**	5
Ye 2018 [15]	***	*	**	6
Yu 2019 [16]	**	*	**	5

Table S3. Bayesian network meta-analysis of operative time in minutes.

OIL		
-11 (-30; 8)	VEIL	
**-74 (-110; -38)**	**-63 (-104; -22)**	RAIL

OIL, open inguinal lymphadenectomy, VEIL, video-endoscopic inguinal lymphadenectomy, RAIL, robot-assisted video-endoscopic inguinal lymphadenectomy.

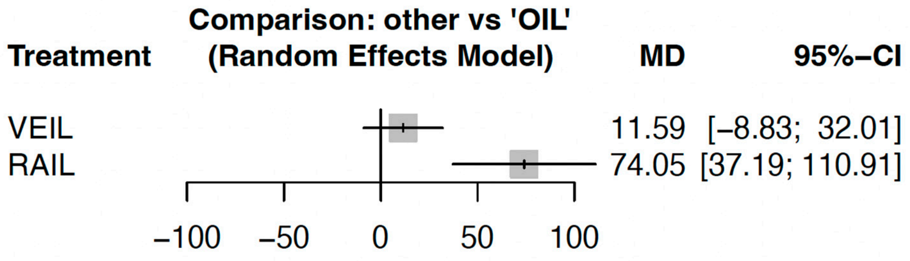

Figure S1. Forest plot of Bayesian network meta-analysis of operative time.

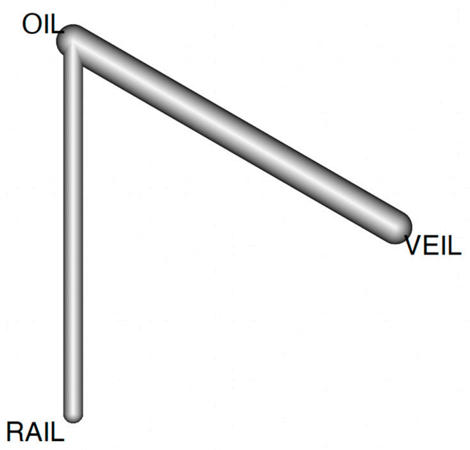

Figure S2. Network graph of Bayesian network meta-analysis of operative time.

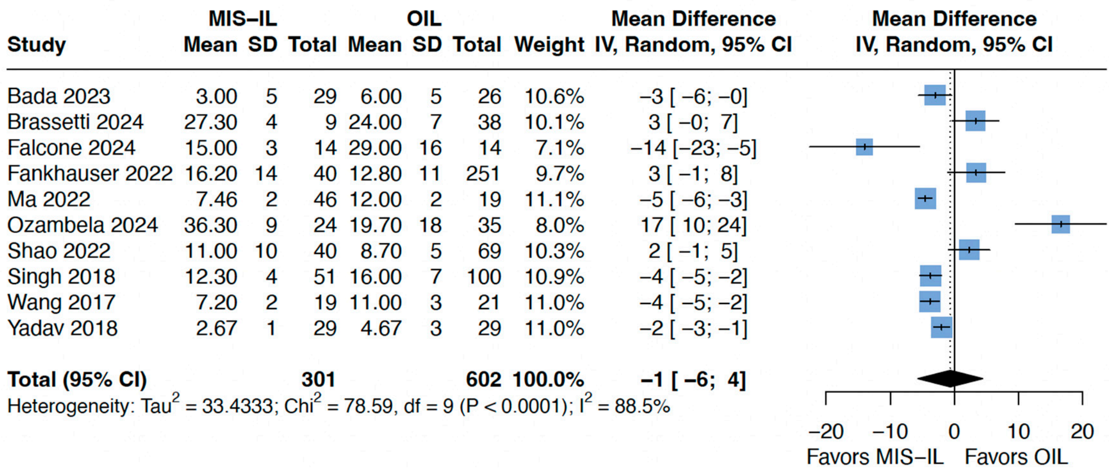

Figure S3. Forest plot comparing O-ILND versus MIS-ILND for time to drain removal [1–4,6,7,9,10,13,14].

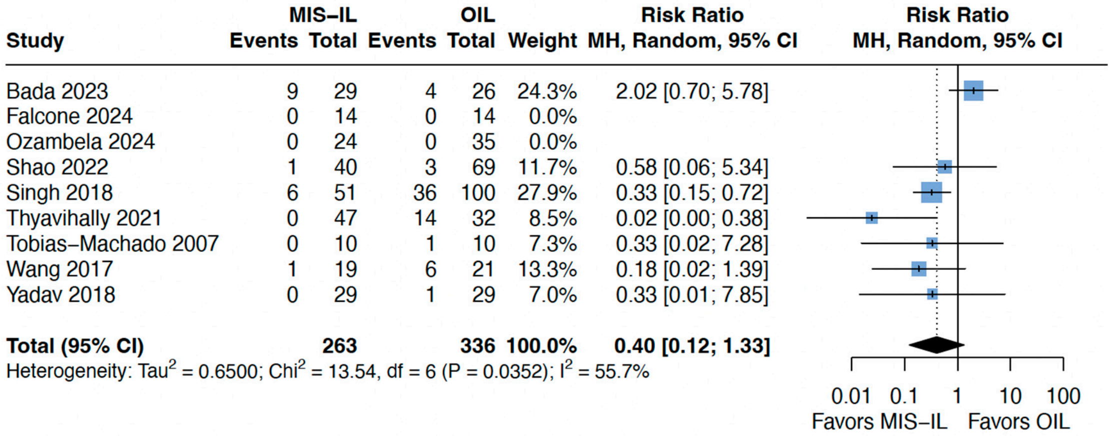

Figure S4. Forest plot comparing O-ILND versus MIS-ILND for skin/flap necrosis [1,3,7,9–14].

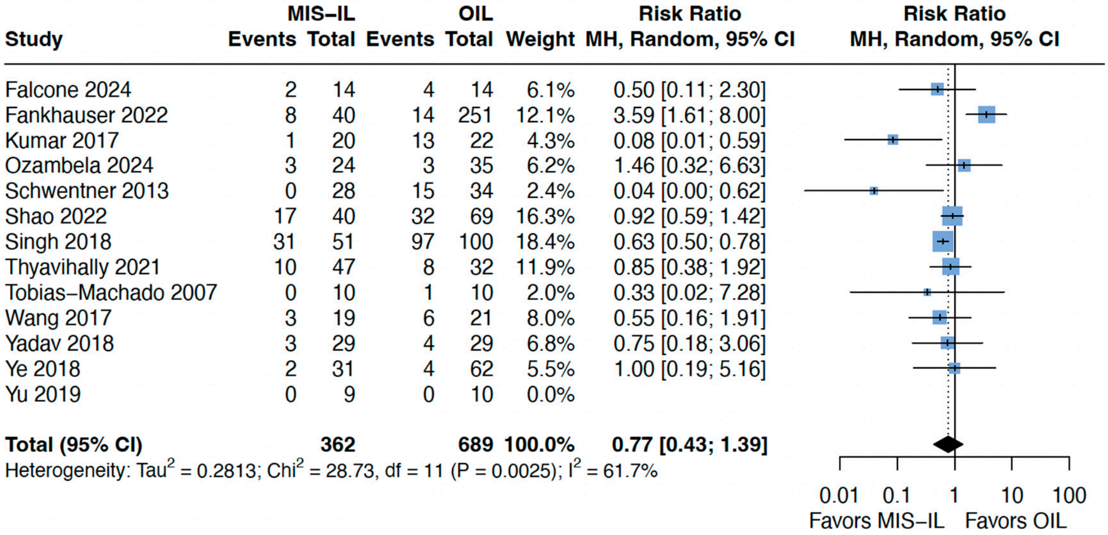

Figure S5. Forest plot comparing O-ILND versus MIS-ILND for lymphedema [3–5,7–16].

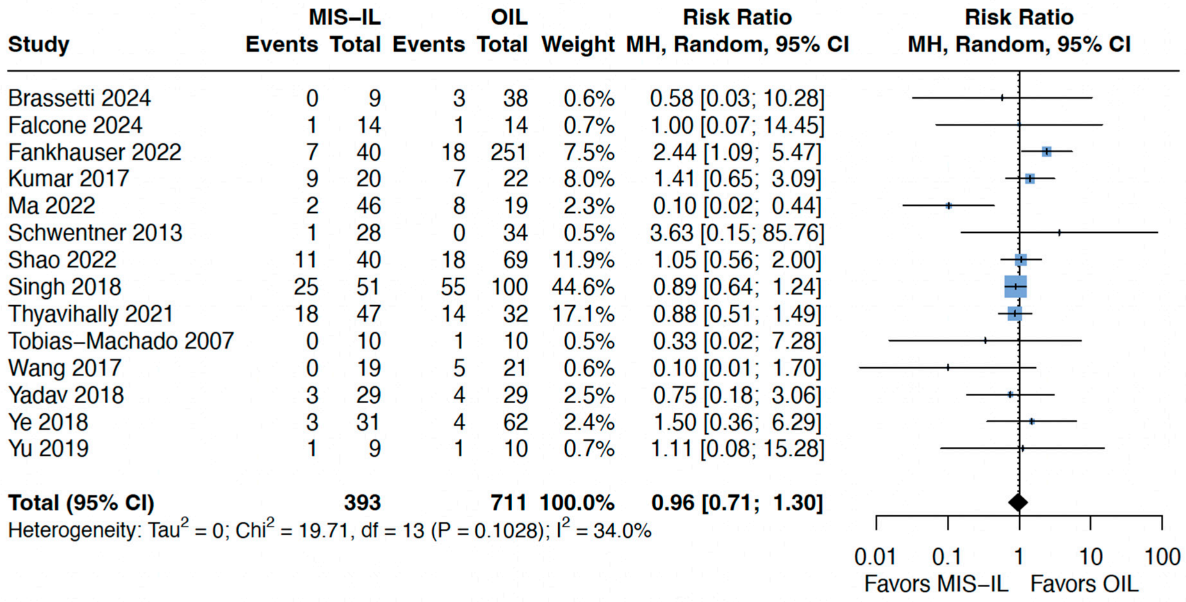

Figure S6. Forest plot comparing O-ILND versus MIS-ILND for lymphocele [2–6,8–16].

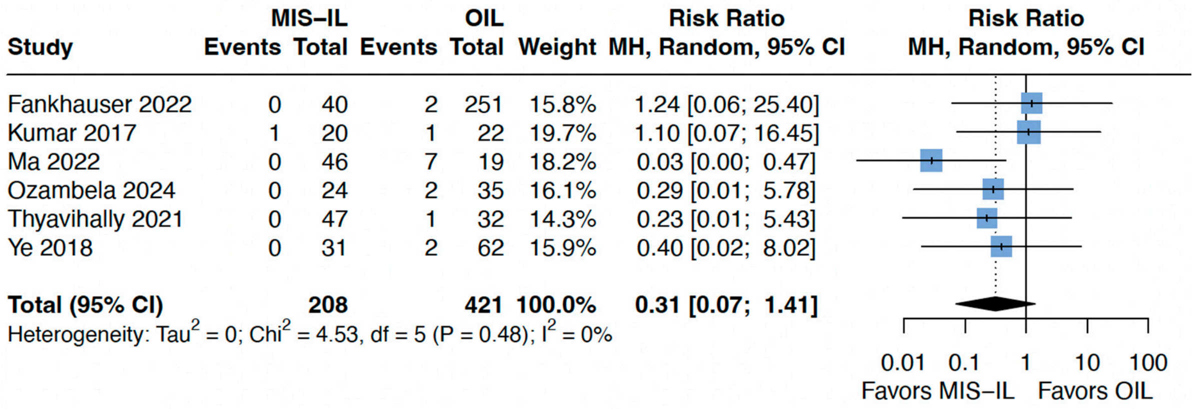

Figure S7. Forest plot comparing O-ILND versus MIS-ILND for deep vein thrombosis [4–7,11,15].

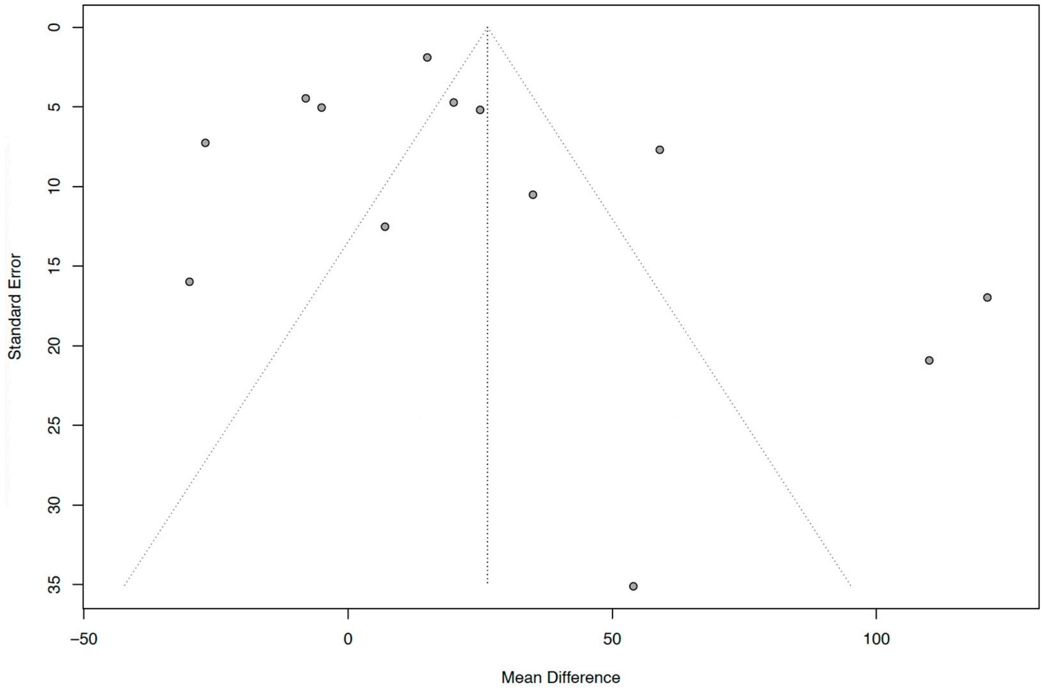

Figure S8. Funnel plot comparing operative time (min).

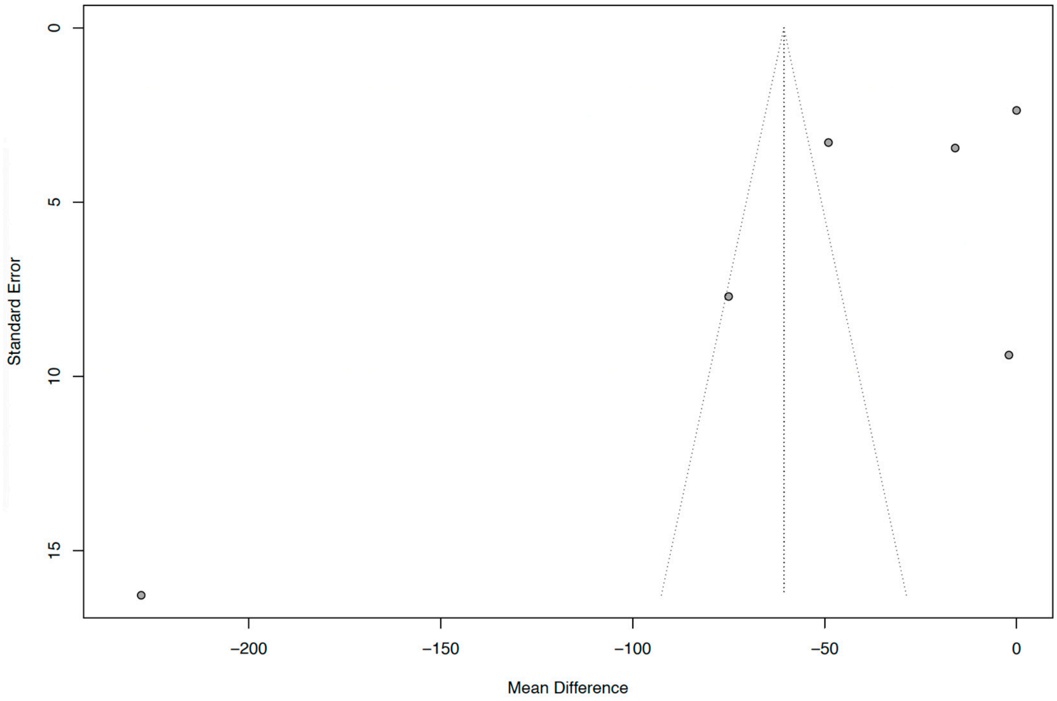

Figure S9. Funnel plot comparing estimated blood loss (mL).

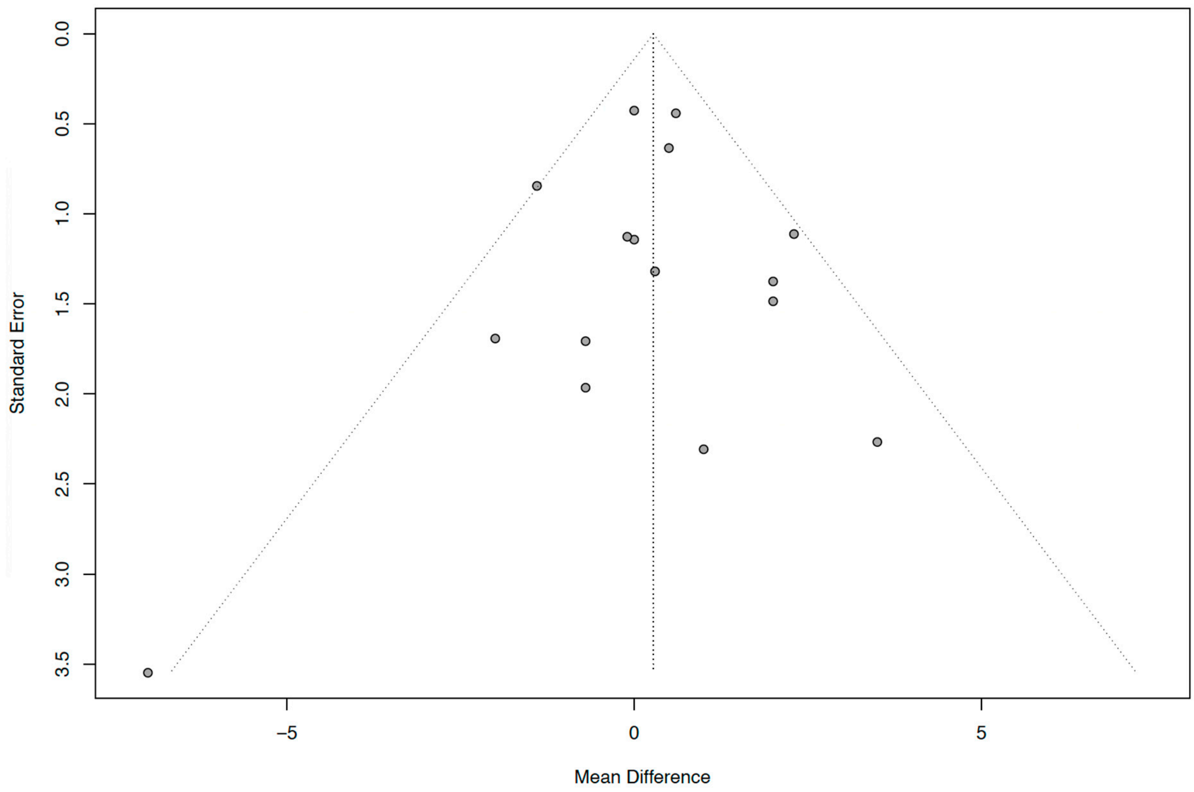

Figure S10. Funnel plot comparing lymph node yield.

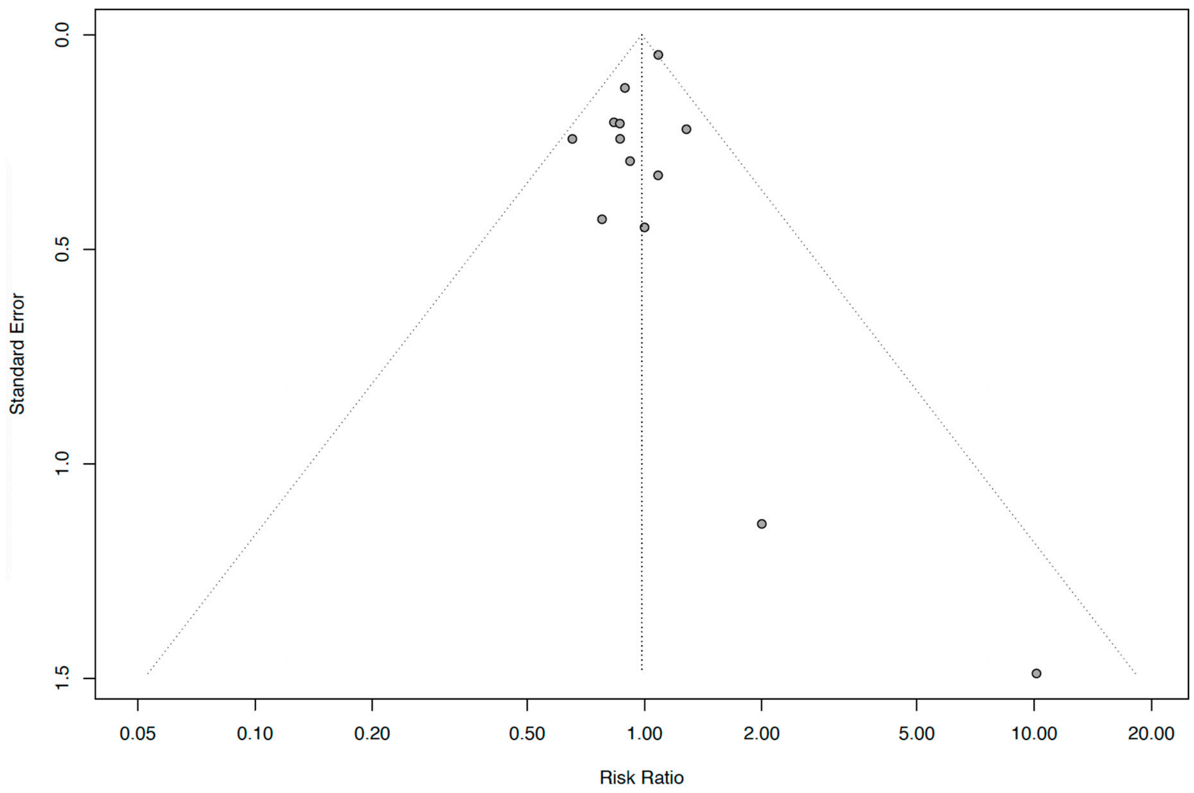

Figure S11. Funnel plot comparing groins with positive inguinal lymph nodes.

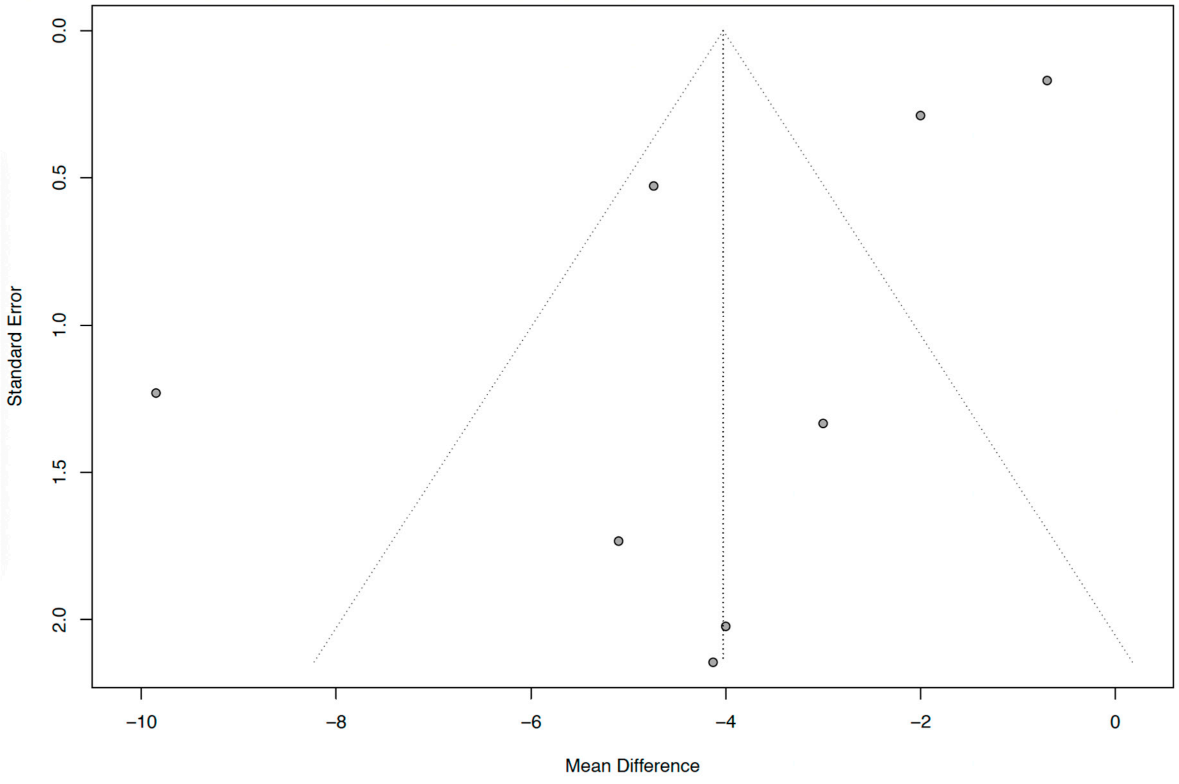

Figure S12. Funnel plot comparing length of stay (days).

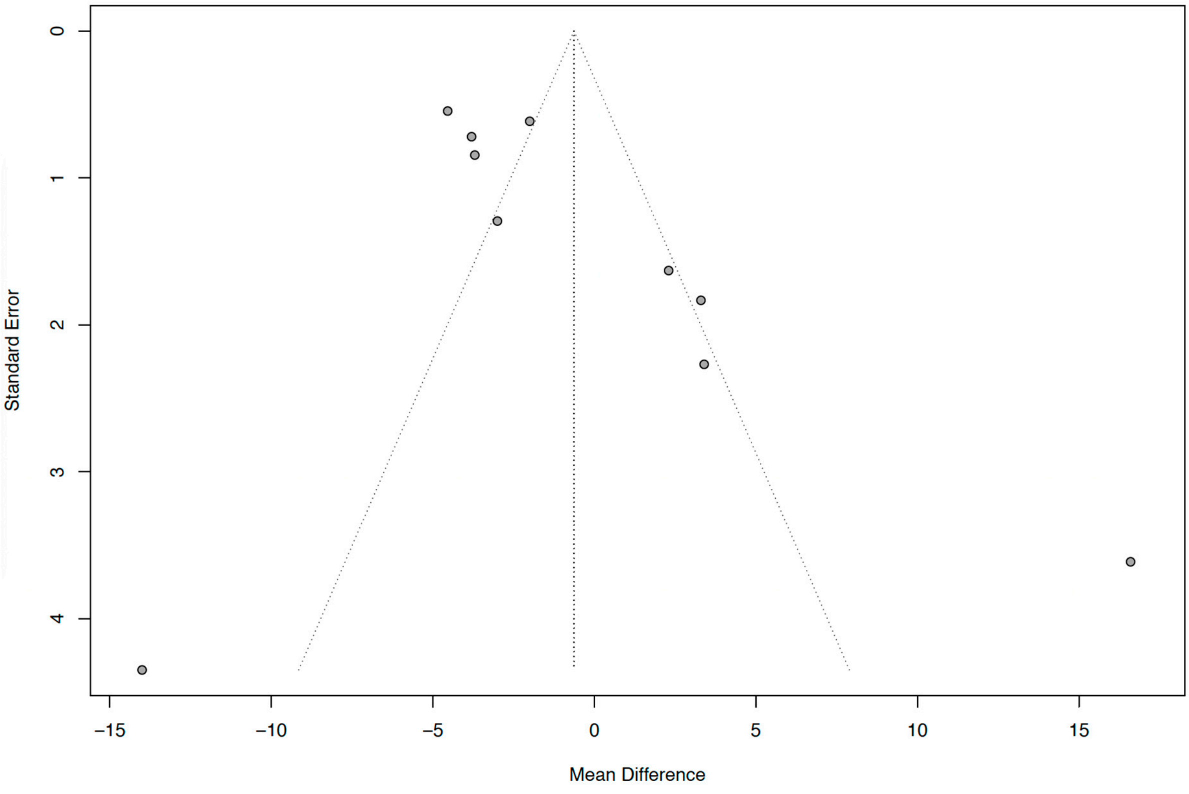

Figure S13. Funnel plot comparing minor complications (Clavien-Dindo Grade 1-2).

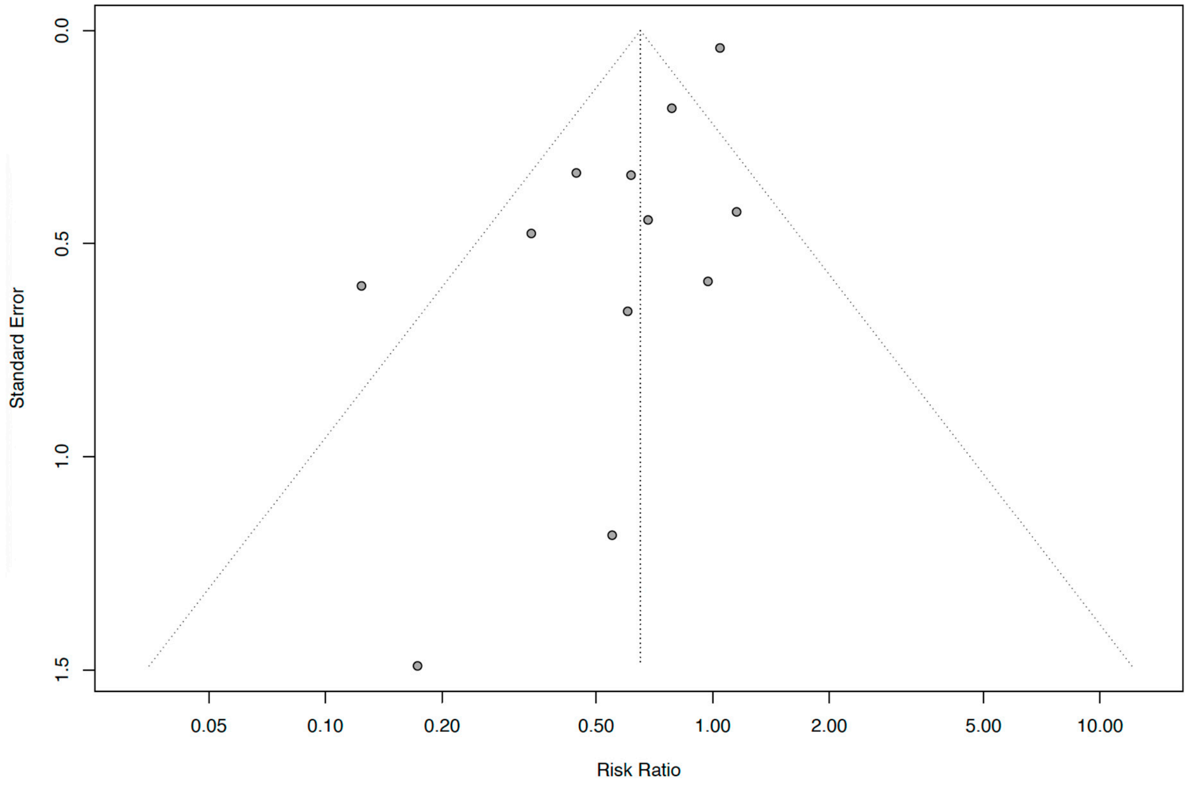

Figure S14. Funnel plot comparing major complications (Clavien-Dindo Grade >2).

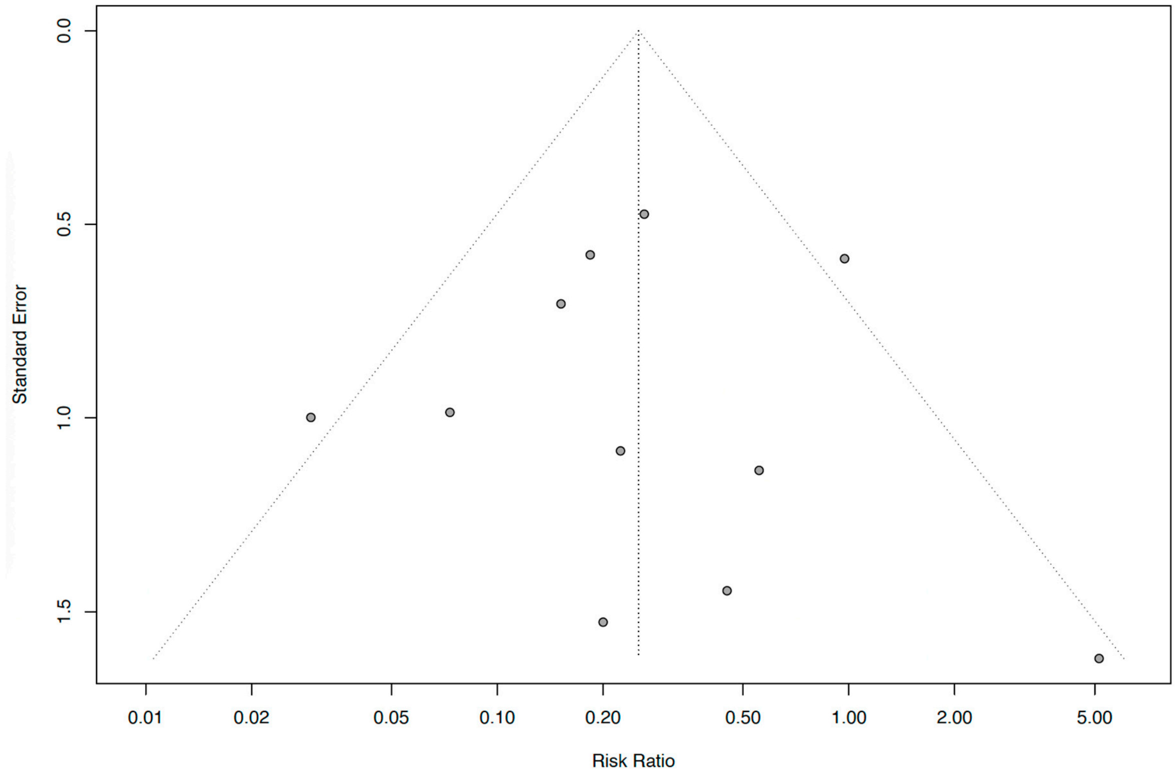

Figure S15. Funnel plot wound infection.

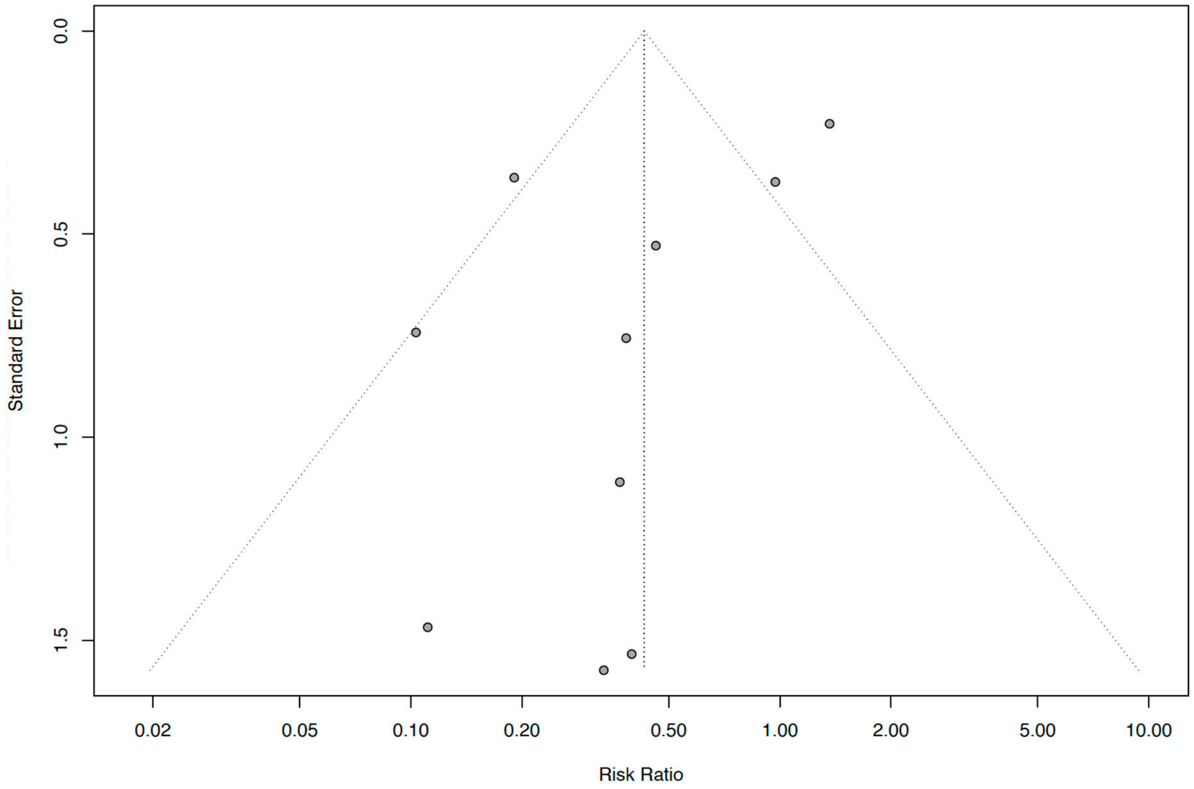

Figure S16. Funnel plot comparing overall recurrence.

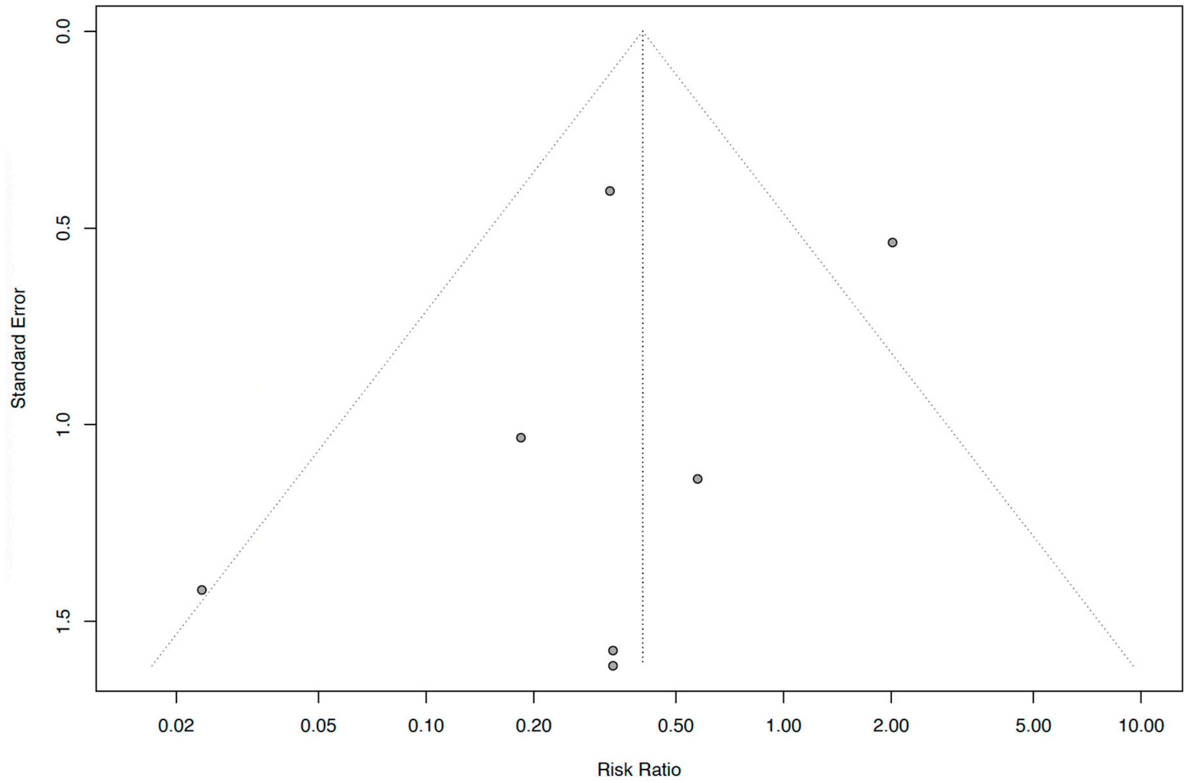

Figure S17. Funnel plot comparing local groin recurrence.

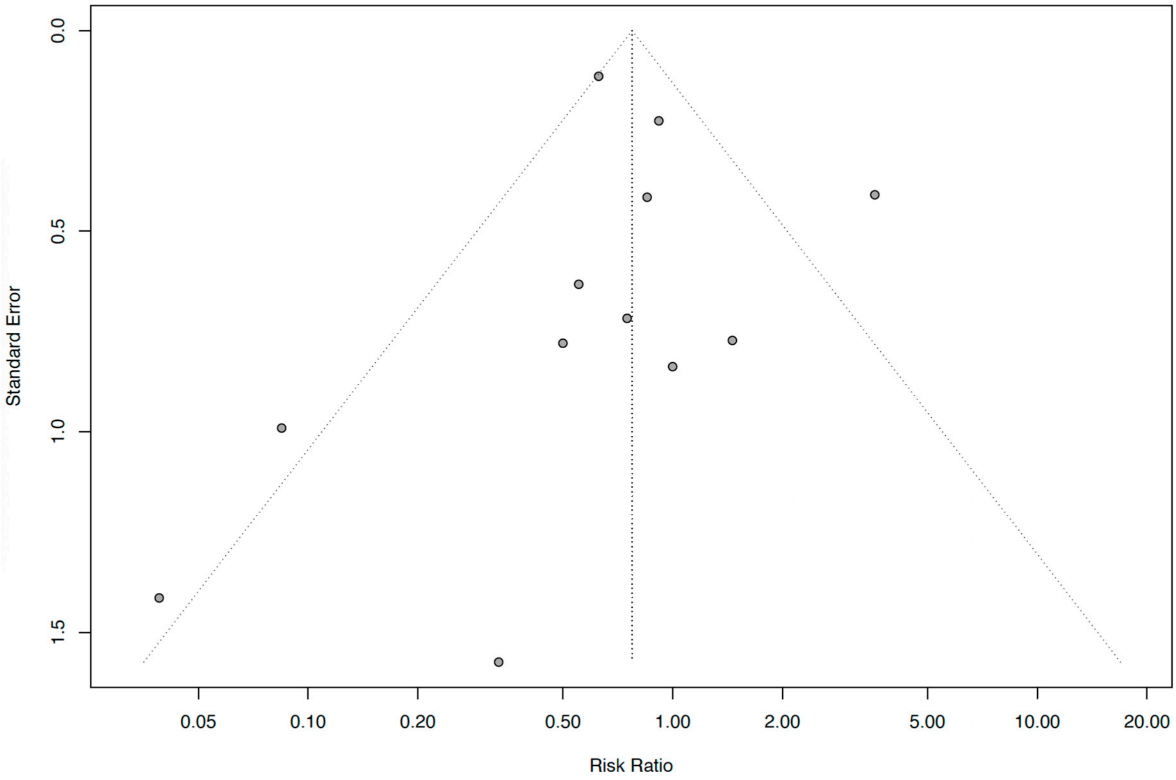

Figure S18. Funnel plot comparing time to drain removal (days).

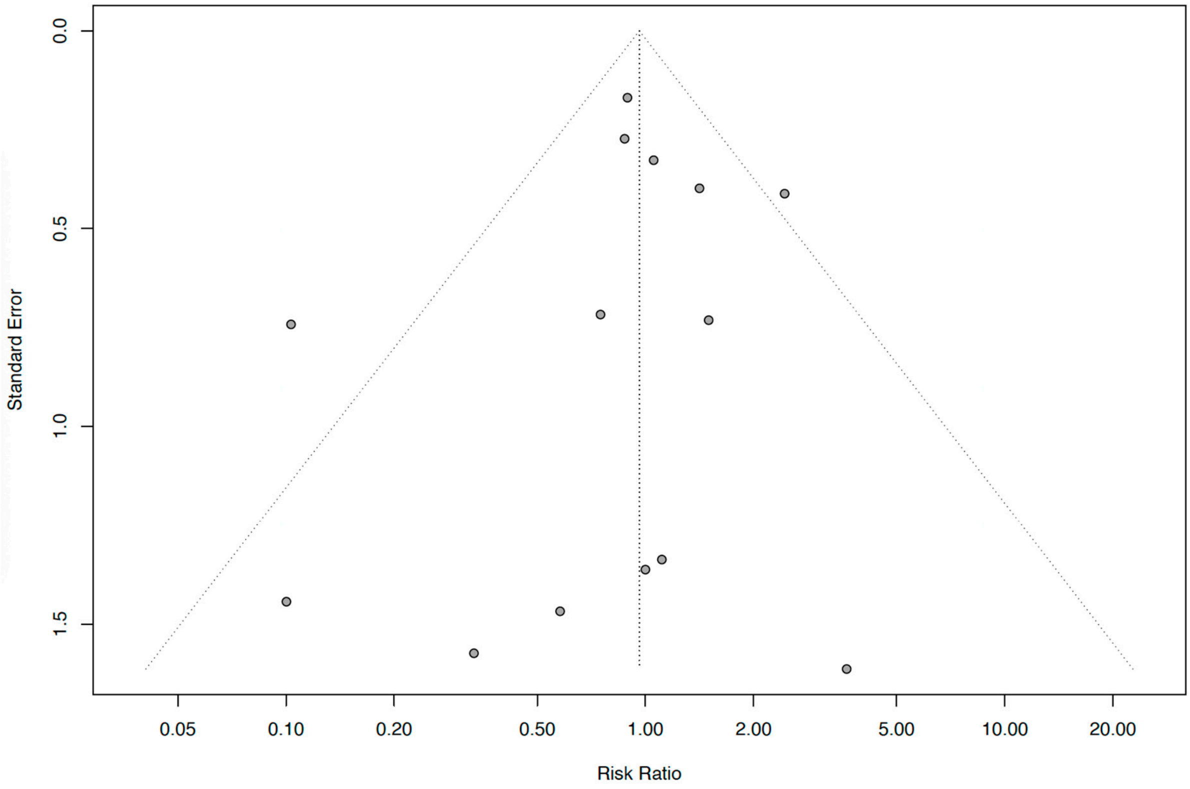

Figure S19. Funnel plot comparing skin/flap necrosis.

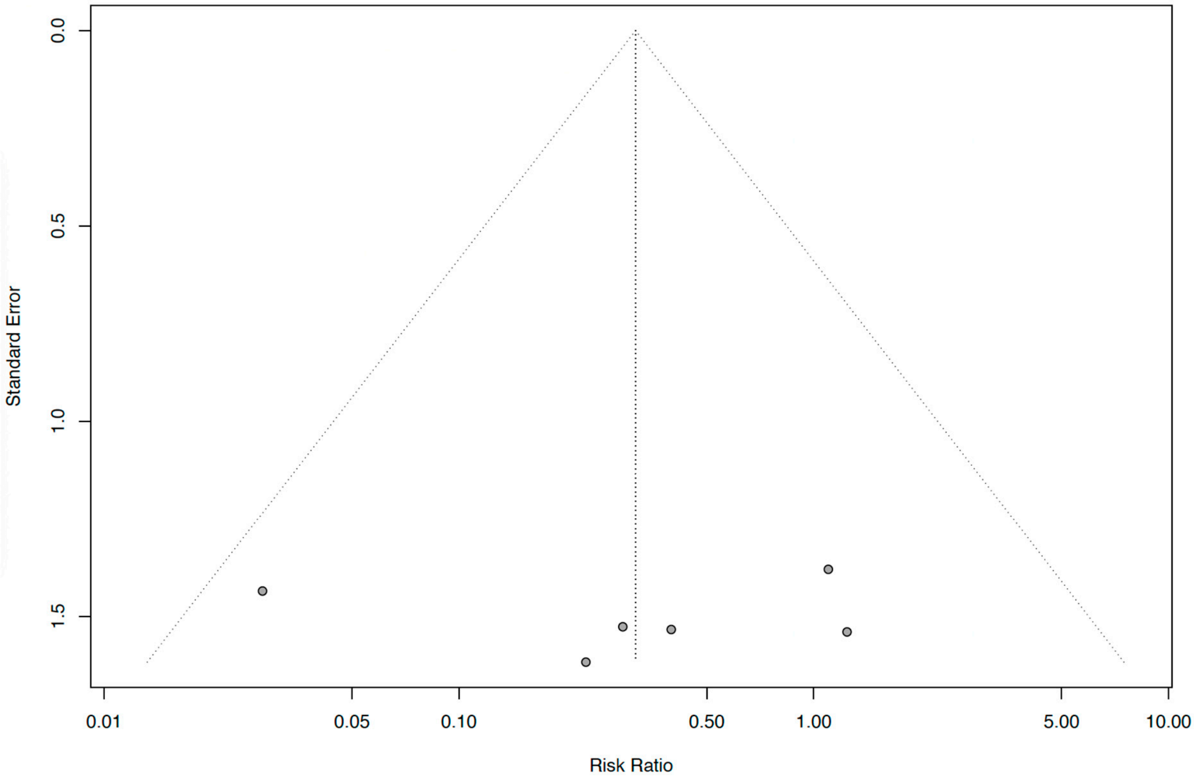

Figure S20. Funnel plot comparing lymphedema.

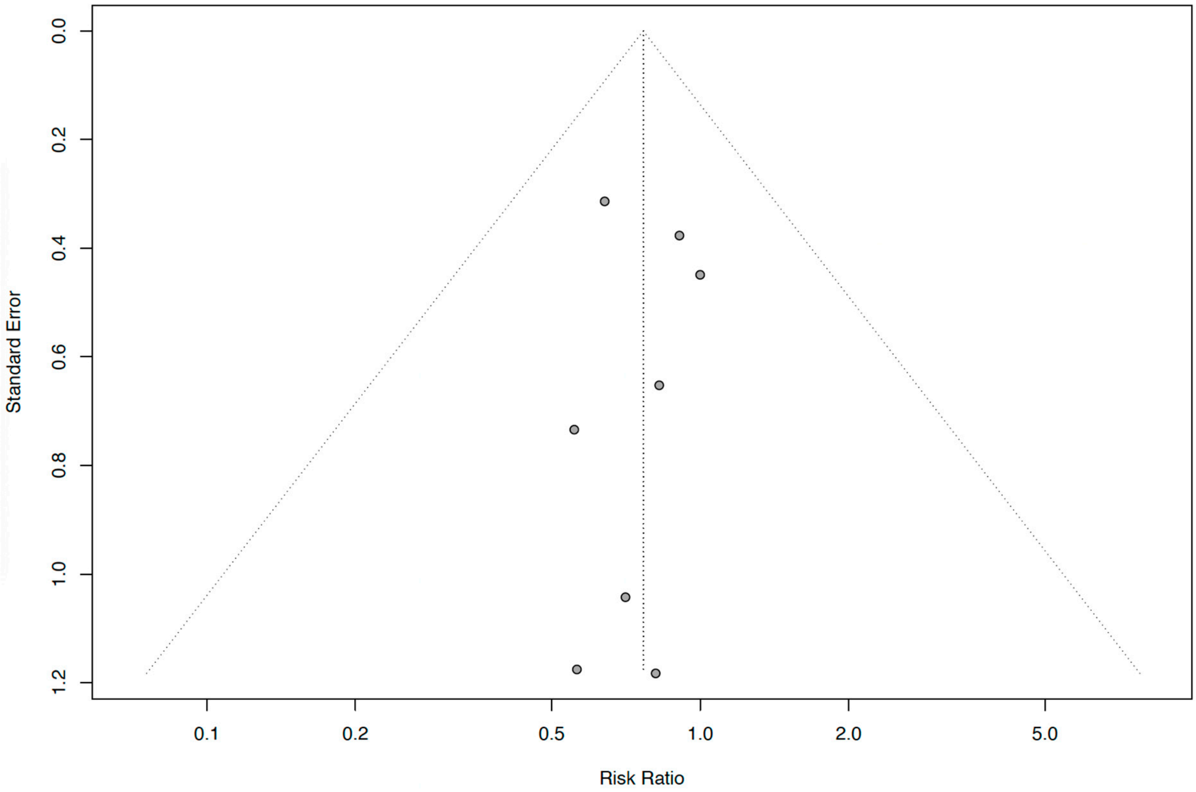

Figure S21. Funnel plot comparing lymphocele.

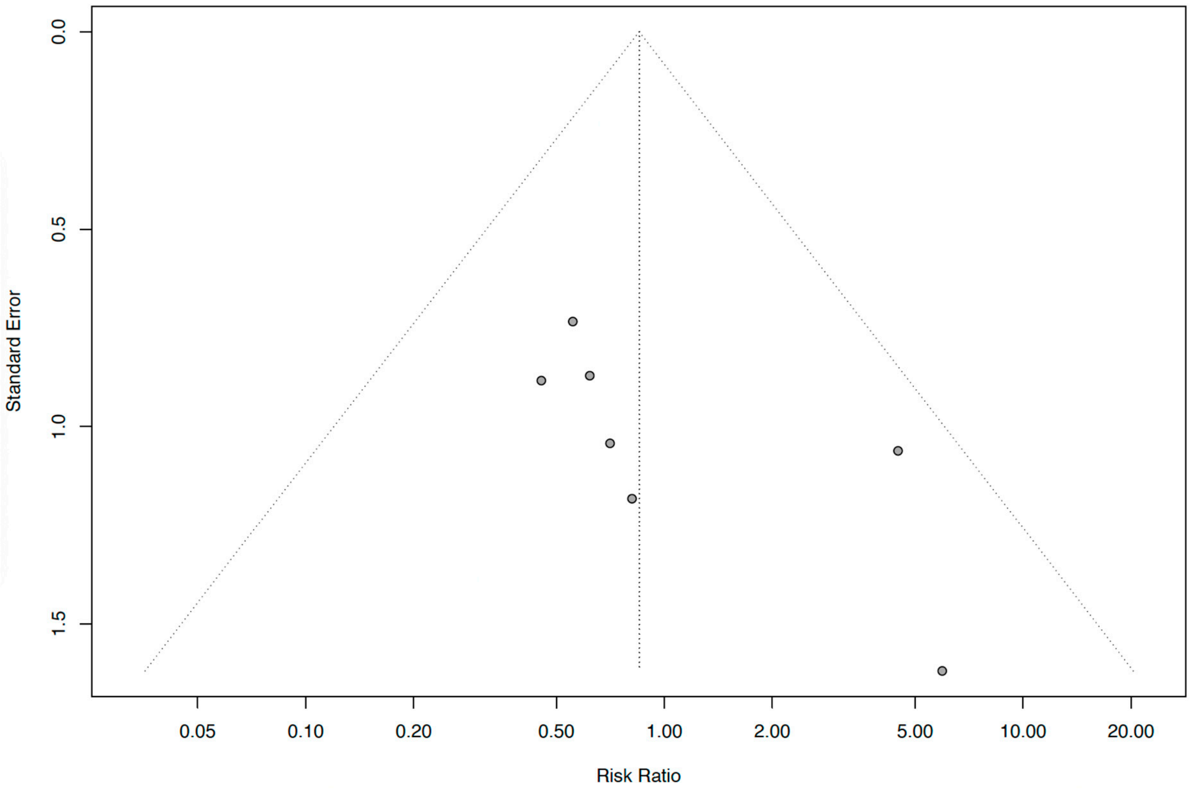

Figure S22. Funnel plot comparing deep vein thrombosis.

Reference

1. Bada, M.; Crocetto, F.; Nyirady, P.; Pagliarulo, V.; Rapisarda, S.; Aliberti, A.; Boccasile, S.; Ferro, M.; Barone, B.; Celia, A. Inguinal lymphadenectomy in penile cancer patients: A comparison between open and video endoscopic approach in a multicenter setting. *J. Basic Clin. Physiol. Pharmacol.* **2023**, *34*, 383–389.
2. Brassetti, A.; Pallares-Mendez, R.; Bove, A.M.; Misuraca, L.; Anceschi, U.; Tuderti, G.; Mastroianni, R.; Licari, L.C.; Bologna, E.; Cartolano, S.; et al. Comparing Outcomes of Open and Robot-Assisted Inguinal Lymphadenectomy for the Treatment of cN2 Squamous Cell Carcinoma of the Penis: A Retrospective Single-Center Analysis. *Cancers* **2024**, *16*, 3921.
3. Falcone, M.; Gül, M.; Peretti, F.; Preto, M.; Cirigliano, L.; Scavone, M.; Sedigh, O.; Oderda, M.; Gontero, P. Inguinal lymphadenectomy for penile cancer: An interim report from a trial comparing open versus videoendoscopic surgery using a within-patient design. *Eur. Urol. Open Sci.* **2024**, *63*, 31–37.
4. Fankhauser, C.D.; Lee, E.W.; Issa, A.; Oliveira, P.; Lau, M.; Sangar, V.; Parnham, A. Saphenous-sparing ascending video endoscopic inguinal lymph node dissection using a leg approach: Surgical technique and perioperative and pathological outcomes. *Eur. Urol. Open Sci.* **2022**, *35*, 9–13.
5. Kumar, V.; Sethia, K.K. Prospective study comparing video-endoscopic radical inguinal lymph node dissection (VEILND) with open radical ILND (OILND) for penile cancer over an 8-year period. *BJU Int.* **2017**, *119*, 530–534.
6. Ma, S.; Zhang, K.; Li, R.; Lu, J.; Wu, T.; Liu, Z.; Fu, X.; Tang, Q.; Ma, J. (Eds.) Bilateral inguinal lymphadenectomy using simultaneous double laparoscopies for penile cancer: A retrospective study. In *Urologic Oncology: Seminars and Original Investigations*; Elsevier: Amsterdam, The Netherlands, 2022.
7. Ozambela, M., Jr.; McCormick, B.Z.; Rudzinski, J.K.; Pieretti, A.C.; González, G.M.N.; Meissner, M.A.; Papadopoulos, J.N.; Adibi, M.; Matin, S.F.; Dahmen, A.S.; et al. (Eds.) Robotic or open superficial inguinal lymph node dissection as staging procedures for clinically node negative high risk penile cancer. In *Urologic Oncology: Seminars and Original Investigations*; Elsevier: Amsterdam, The Netherlands, 2024.
8. Schwentner, C.; Todenhöfer, T.; Seibold, J.; Alloussi, S.H.; Mischinger, J.; Aufderklamm, S.; Stenzl, A.; Gakis, G. Endoscopic inguinofemoral lymphadenectomy—Extended follow-up. *J. Endourol.* **2013**, *27*, 497–503.
9. Shao, Y.; Hu, X.; Ren, S.; Liao, D.; Yang, Z.; Liu, Y.; Lia, T.; Wu, K.; Xiong, S.; Yang, W.; et al. Comparison of different surgical methods and strategies for inguinal lymph node dissection in patients with penile cancer. *Sci. Rep.* **2022**, *12*, 2560.

10. Singh, A.; Jaipuria, J.; Goel, A.; Shah, S.; Bhardwaj, R.; Baidya, S.; Jain, J.; Jain, C.; Rawal, S. Comparing Outcomes of Robotic and Open Inguinal Lymph Node Dissection in Patients with Carcinoma of the Penis. *J. Urol.* **2018**, *199*, 1518–1525.
11. Thyavihally, Y.B.; Dev, P.; Waigankar, S.S.; Pednekar, A.; Kulkarni, B.; Sharma, A.; Maheshwari, S.; Roy, D.; Agarwal, V.; Khandekar, A.A.; et al. Comparative study of perioperative and survival outcomes after video endoscopic inguinal lymphadenectomy (VEIL) and open inguinal lymph node dissection (O-ILND) in the management of inguinal lymph nodes in carcinoma of the penis. *J. Robot. Surg.* **2021**, *15*, 905–914.
12. Tobias-Machado, M.; Tavares, A.; Silva, M.N.R.; Molina, J.W.R.; Forseto, P.H.; Juliano, R.V.; Wroclawski, E.R. Can Video Endoscopic Inguinal Lymphadenectomy Achieve a Lower Morbidity Than Open Lymph Node Dissection in Penile Cancer Patients? *J. Endourol.* **2008**, *22*, 1687–1692.
13. Wang, S.; Du, P.; Tang, X.; An, C.; Zhang, N.; Yang, Y. Comparison of Efficiency of Video Endoscopy and Open inguinal lymph node dissection. *Anticancer. Res.* **2017**, *37*, 4623–4628.
14. Yadav, S.S.; Tomar, V.; Bhattar, R.; Jha, A.K.; Priyadarshi, S. Video Endoscopic Inguinal Lymphadenectomy vs Open Inguinal Lymphadenectomy for Carcinoma Penis: Expanding Role and Comparison of Outcomes. *Urology* **2018**, *113*, 79–84.
15. Ye, Y.L.; Guo, S.J.; Li, Z.S.; Yao, K.; Chen, D.; Wang, Y.J.; Chen, P.; Han, H.; Zhou, F.J. Radical videoscopic inguinal lymphadenectomies: A matched pair analysis. *J. Endourol.* **2018**, *32*, 955–960.
16. Yu, H.; Lu, Y.; Xiao, Y.; Guo, J.; Yin, X.; Yang, Y.; Wang, H.; Gao, J. Robot-assisted laparoscopic antegrade versus open inguinal lymphadenectomy: A retrospective controlled study. *BMC Urol.* **2019**, *19*, 135.
